# Long-term effects of an egg-protein hydrolysate on cognitive performance and brain vascular function: a double-blind randomized controlled trial in adults with elevated subjective cognitive failures

**DOI:** 10.1007/s00394-024-03394-y

**Published:** 2024-05-04

**Authors:** Micah S. Adams, Ronald P. Mensink, Jogchum Plat, Peter J. Joris

**Affiliations:** Department of Nutrition and Movement Sciences, NUTRIM Institute of Nutrition and Translational Research in Metabolism, Maastricht University Medical Center+, Universiteitssingel 50, P.O. Box 616, 6200 MD Maastricht, The Netherlands

**Keywords:** Brain vascular function, Cerebral blood flow, Cognitive performance, Egg-protein hydrolysates, Subjective cognitive failures

## Abstract

**Purpose:**

Short-term intake of the egg-protein hydrolysate Newtricious (NWT)-03 improved executive function, but underlying mechanisms and long-term effects, including other cognitive domains, are unknown.

**Methods:**

A 36-week randomized controlled trial involving 44 overweight/obese individuals experiencing elevated Subjective Cognitive Failures (SCF; aged 60–75 years) assessed the impact of daily consumption of 5.7 g of NWT-03 or placebo powders on cognitive performance (psychomotor speed, executive function, memory) and Cerebral Blood Flow (CBF), a marker of brain vascular function. Cognitive performance was evaluated using a neurophysiological test battery (CANTAB) and CBF was measured using magnetic resonance imaging perfusion method Arterial Spin Labeling (ASL). Serum samples were collected to determine brain-derived neurotrophic factor (BDNF) concentrations.

**Results:**

Anthropometrics, and energy and nutrient intakes remained stable throughout the trial. NWT-03 was well tolerated, and compliance was excellent (median: 99%; range: 87–103%). No overall intervention effects were observed on cognitive performance or CBF, but post-hoc analyses revealed significant improvements on executive function in women, but not men. Specifically, a reduction of 74 ms in reaction latency on the multitasking task (95% CI: −134 to −15; *p* = 0.02), a reduction of 9 between errors (95%CI: −14 to −3; *p* < 0.001), and a reduction of 9 total errors (95%CI: −15 to −3; *p* < 0.001) on the spatial working memory task were found in women. No intervention effects were observed on serum BDNF concentrations (*p* = 0.31).

**Conclusion:**

Long-term consumption of NWT-03 improved multitasking abilities and working memory in women with elevated SCF. Brain vascular function remained unaffected. Sex differences in executive function require additional clarification.

**Supplementary Information:**

The online version contains supplementary material available at 10.1007/s00394-024-03394-y.

## Introduction

People with elevated Subjective Cognitive Failures (SCF), which are defined as the self-awareness of having brief failures in memory, perception, and/or action causing errors [1, 2], may be indicative of an increased risk of developing dementia [3]. Increased attention has been drawn to dietary interventions as a means to limit the progression of cognitive decline and to prevent the long-term development of dementia [4]. In fact, there are already indications that long-term dietary protein and protein hydrolysate intake improves cognitive performance, particularly in attention and psychomotor speed [5–10] and on executive function domains [7, 11–16]. Moreover, evidence already exists for the beneficial effects of egg-protein hydrolysates on cognitive performance. For instance, Mohajeri and colleagues have revealed in their study involving middle-aged women that 1.0 g/day of a tryptophan-rich egg-protein hydrolysate for 19 days improved executive function based on the Go/No-Go task [7]. Furthermore, the effects of Newtricious (NWT-03), another egg-protein hydrolysate derived from lysozyme hydrolysis from egg whites, were recently evaluated [17]. In a 4-week intervention trial in men and women with metabolic syndrome, we have shown that daily intake of 5.0 g NWT-03 also improved cognitive performance in the executive function domain based on the anti-cue reaction time task [18], with no effects on psychomotor speed.

The mechanisms underlying the observed improvements in cognitive performance after consuming protein hydrolysates are not well understood. Some suggestions indicate that NWT-03 may enhance peripheral vascular function [19] and cardiometabolic risk markers [20]. However, it is unknown if these effects translate to the brain, as impaired brain vascular function is considered an important mechanism preceding the development of an impaired cognitive performance [21, 22]. Previous randomized controlled trials (RCTs) have reported that dietary protein and protein hydrolysate interventions may enhance brain vascular function, as evidenced by improvements in cerebral blood flow (CBF) [6, 11, 23–25]. However, the effects of egg-protein hydrolysates on CBF have never been studied before. Additionally, the long-term maintenance of these cognitive performance benefits and their extension to other cognitive domains remain to be established. Therefore, the primary objective of this double-blind, randomized, placebo-controlled parallel study was to investigate long-term (36 weeks) effects of an NWT-03 intervention on cognitive performance and CBF, as quantified by the non-invasive magnetic resonance imaging (MRI) perfusion method Arterial Spin Labeling (ASL). Furthermore, we investigated effects on an additional cognitive domain, namely memory, which was assessed using the Cambridge Neuropsychological Test Battery (CANTAB). The intervention specifically targeted older adults experiencing elevated SCF, as they may benefit most from intervention strategies.

## Materials and methods

### Study participants

Forty-four older men and women aged between 60 and 75 with elevated SCF were recruited using online advertisements, posters in public buildings within Maastricht, and among participants who had taken part in prior studies. Interested candidates were invited for a screening visit to the university if they indicated that they had a BMI between 25 and 35 kg/m^2^ (overweight or obese), were right-handed, did not have any chronic medical conditions such as diabetes, hypertension, and active cardiovascular disease (CVD), and noticed that they were experiencing symptoms of cognitive decline. Anthropometrics, blood pressure, and fasting blood samples were taken during the screening visit. We utilized the Cognitive Failure Questionnaire (CFQ) [1, 26] to assess the presence of elevated SCF. This 25-item questionnaire evaluates the frequency of everyday cognitive errors based on a score of 1 (very often) to 5 (never). A cut-off value of ≥ 40 was used to define elevated SCF, which corresponds to approximately 23% of the older population [26]. In addition to the foregoing criteria, included participants needed to have a fasting plasma glucose < 7.0 mmol/L, serum total cholesterol < 8 mmol/L, serum triacylglycerol < 4.5 mmol/L, systolic blood pressure < 160 mmHg, diastolic blood pressure < 100 mmHg and a stable body weight. They also had to be willing to give up being a blood donor 8 weeks before and during the study, and 4 weeks after completion. Based on a questionnaire, they also needed to indicate that they did not possess any contraindications for an MRI scan. All study participants provided written informed consent before the start of the screening visit, and the study protocol was approved by the Medical Ethical Review Committee of the Maastricht University Hospital and Maastricht University (METC azM/UM) (NL75618.068.20) and registered at ClinicalTrials.gov in January 2021 as NCT04831203. This study has been performed in accordance with the ethical standards laid down in the 1964 Declaration of Helsinki and its later amendments and was conducted from May 2021 until October 2022.

### Study design

A 36-week double-blind, randomized, controlled parallel study was performed. The randomization was performed by an independent researcher using WinPepi Etcetera software, stratified for sex. The sachets were packaged in numbered boxes, aligning with a randomization process of which the investigators were unaware. These boxes were then dispensed by M.S.A. All study outcomes were performed at two baseline test days and identical follow-up visits. These measurements included anthropometrics (height, weight, body mass index [BMI], waist and hip circumferences, and skinfold measurements to determine body fat percentage [27]), cognitive performance (CANTAB) and brain vascular function (CBF). At weeks 9, 18, and 27, the participants revisited the university to obtain additional supplies and to return the empty sachets in order to check compliance. Moreover, food frequency questionnaires (FFQ) were filled out during baseline and follow-up visits to evaluate dietary intake during the previous 4 weeks [28]. They were instructed not to perform any strenuous physical exercise or consume alcoholic beverages 48 h before the test days, and to fast for 12 h before blood sampling. Moreover, to standardize measurements, participants were asked to come to the university by public transport or car instead of walking or biking. All measurements were performed by the same investigator (M.S.A.), at the same time of day, and at the same location (Metabolic Research Unit [MRUM] and the Scannexus research facilities in Maastricht). Finally, study participants were requested to record in study diaries any protocol deviations or changes in their health status, medication use, and alcohol intake.

### Study products

Participants either received 5.7 g of the egg-protein hydrolysate product (NWT-03; Newtricious R&D, ‘s-Hertogenbosch, The Netherlands) or 5.7 g of a maltodextrin placebo in dry powder sachets to mix with 200 mL of water and to consume every day in the morning before breakfast. In addition, the spray-dried egg-protein hydrolysate (NWT-03; Nizo Food Research, Ede, The Netherlands) contained citric acid, flavoring, acesulfame K, sucralose, and quinine HCL. The matching placebo consisted of maltodextrin from potato starch, flavoring, citric acid, cloudifier, tartaric acid, malic acid, acesulfame K, sucralose, caramel E150a, and quinine HCL. The products were similar in color and taste (lemon flavor) to ensure the double-blind research design and packaged in a box that was labeled according to Good Manufacturing Process (GMP) guidelines.

### Cognitive performance

In line with our prior studies [6, 29], cognitive performance was assessed using the fully automated CANTAB software on a digital touchscreen tablet (iPad, 5th generation; Apple). Written and verbal instructions were provided by the investigator to familiarize the participants with the tablet before proceeding to the cognitive tasks. In addition, the software offered further guidance and practice trials to ensure their understanding and proficiency in using the tablet.

To investigate psychomotor speed, the reaction time task (RTI) was performed, and the resulting reaction time (ms) and movement time (ms) were evaluated as outcome parameters. The executive function domain was appraised using the multitasking task (MTT) with incongruency cost (ms), median reaction latency (ms), multitasking cost (ms), and total incorrect errors as the key outcomes. Furthermore, the spatial working memory (SWM) task additionally served to evaluate executive function by analyzing between errors, total errors, and strategy scores. Lastly, the memory domain was examined with a delayed matching to sample (DMS) and paired associates learning (PAL) task. The percentage of correctly answered trials for all delays specified DMS results, whereas PAL was evaluated based on total errors and the first attempt memory score. A more detailed description of these tests has previously been provided [6].

### Brain vascular function

MRI with ASL scans were performed on a Siemens 3.0 Tesla Magnetom Prisma Fit scanner with a 64-channel head coil. Detailed information regarding the acquisition and processing of MRI data has formerly been published [6, 29]. In short, participants remained in the supine position for an approximately 20-min acclimatization period during which an MPRAGE (T1 image) structural scan was acquired, and a labeling plane was situated based on an angiogram perpendicular to the vertebral and carotid arteries. Afterwards, pseudo-continuous arterial spin labeling (pCASL) with spin echo readouts and background suppressed segmented 3-D gradients were performed. Over the course of the 9-min sequence, parameters included a TR 4050 (repetition time), TE 13.6 ms (echo time), GRAPPA 2, labelling duration 1750 ms, post-labelling delay 2000 ms, and ten label-control repetitions.

Motion correction was automatically performed on the Siemens scanner. To perform brain extraction and tissue segmentation for the anatomical MPRAGE image, Volbrain [30] was used. Quantitative CBF images were estimated from the ASL data using FSL software (Version 6.0) [31], and perfusion-weighted images were generated by pairwise subtraction of label and control images on the pCASL data. Based on the recommendations of the ASL White Paper [32], the BASIL tool (v 4.0.15) [33] quantified perfusion-weighted images. Using boundary-based registration to the brain extracted MPRAGE image by the FLIRT routine [34], the calibrated ASL images with absolute CBF values were co-registered. Gray matter, global brain, and cortical and subcortical CBF values were also determined using the MPRAGE scan. Subsequently, voxel-wise comparisons [35] were conducted by first registering the absolute CBF values to MNI (2 mm) space using a nonlinear algorithm (FNIRT). Due to a high signal-to-noise ratio (SNR), smoothing was not performed and nonparametric permutation testing with a threshold-free cluster enhancement within FSL was used to determine significantly different clusters. These clusters were determined from applying an unpaired t-task with a single-group a paired difference (FMRIB’s Local Analysis of Mixed Effects stage 1 (FLAME) [36]), voxel connectivity of 26, and a z-threshold of 2.3 (*p* value < 0.05). Based on the smoothness estimates, family-wise corrections for multiple comparisons were performed. The Harvard–Oxford subcortical structural atlas and Atlasquery function were used to determine the average probability of the cluster locations.

### Brain-derived neurotrophic factor

At baseline and follow-up visits, fasting serum samples were collected using venipuncture on the forearm. Samples were centrifuged at 1300 × g for 10 min at 21 °C after clotting for approximately 60 min, aliquoted, frozen in liquid nitrogen, and stored at −80 °C. At the end of the study, all samples were analyzed for brain-derived neurotrophic factor (BDNF) concentrations by an enzyme-linked immunosorbent assay (Duo Kit ELISA, R&D Systems, Minneapolis, USA) according to manufacturer’s instructions. The results are reported as the sum of precursor BDNF and mature BDNF because the assay did not differentiate between them.

### Statistical analyses

Cognitive performance and CBF were the primary study outcomes for this NWT-03 intervention. Power calculations were conducted for an analysis of covariance (ANCOVA) with baseline measures of the outcomes as a covariate. It was calculated that 18 participants per group were needed to detect clinically relevant changes of at least 5% in reaction latency within the executive function domain (MTT task). This power calculation was based on our prior trial using a between-subject variability of 10%, correlation coefficient of 0.75 [29], > 80% power, and two-sided alpha of 0.05. Moreover, a sample size of 18 participants per group was also sufficient to reach a power >80% to detect changes in CBF of at least 10%, using a between-subject variability of 18%, correlation coefficient of 0.68 [37–39], and two-sided alpha of 0.05.

Using the Shapiro–Wilk test, all variables were normally distributed. A one-way ANCOVA, using the baseline measurements of the outcome variables as covariates and intervention as a fixed factor was conducted to determine differences in responses between intervention and placebo groups. In preliminary analyses, we incorporated age as a potential effect-modifier into the ANCOVA model. However, this factor did not affect the outcomes and was, therefore, omitted from the final model. Furthermore, post-hoc analyses using a sex × intervention interaction as a fixed factor were also performed to test for differential effects between men and women. If the interaction was significant, then we separated the men and women and reran the analysis. If it was not significant, then we removed the interaction term from the final model. All statistical analyses were performed using SPSS (IBM Corp., IBM SPSS Statistics, V27, Armonk, NY, USA) and differences were deemed statistically significant using two-tailed tests at *p* ≤ 0.05.

## Results

### Study participants

**Fig. 1:**
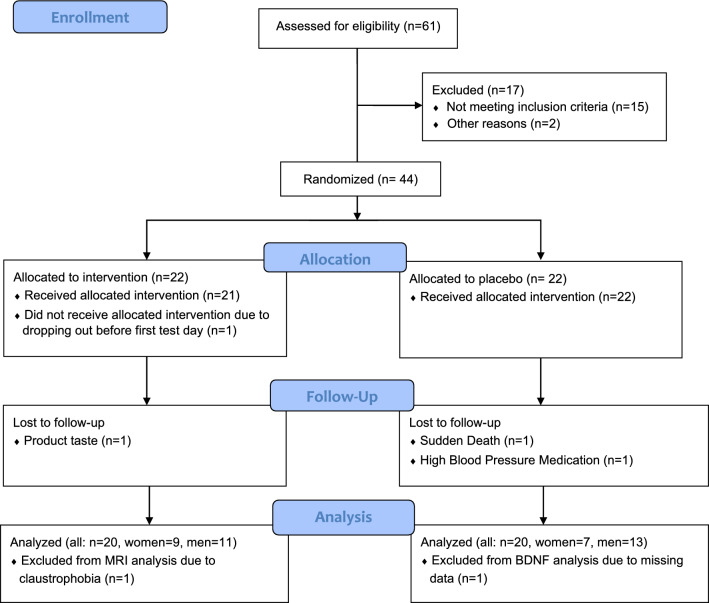
Illustrates a Consolidated Standards of Reporting Trials (CONSORT) flow diagram. A total of forty-four eligible men and women were randomized into intervention or placebo groups. A total of 40 participants completed the study and were included in the statistical analysis. One participant was excluded from the MRI analysis due to experiencing claustrophobia on the follow-up test day. Another participant was excluded from the BDNF analysis due to a missing value at baseline

A Consolidated Standards of Reporting Trials (CONSORT) flow diagram is shown in Fig. 1. Forty-four eligible men (*n* = 26) and women (*n* = 18) were randomized into the intervention or placebo groups. The mean age of the participants was 69 ± 4 years, and their average BMI was 28.1 ± 2.6 kg/m^2^ (Table 1). Two people allocated to the intervention dropped out for personal reasons or because they disliked the taste of the product. An additional participant allocated to the placebo group dropped out due to starting high blood pressure medication. Finally, a fatal serious adverse event occurred in one participant who was consuming the placebo, which was approved by the METC azM/UM as unrelated to the ongoing investigation. Twenty participants in the intervention (*n* = 9 women, *n* = 11 men) and twenty participants in the placebo group completed the placebo (*n* = 7 women, *n* = 13 men) completed the study and were included in the analyses. After experiencing claustrophobia on the follow-up test day, one participant had missing CBF data. Additionally, one person was excluded from the BDNF analysis due to a missing value at baseline. No further serious adverse events or protocol deviations were reported in the study diaries and the study product was well tolerated. Compliance based on empty returned sachets was excellent (median: 99%; range: 87–103%). Anthropometrics including body weight, BMI, and body fat percentages derived from skinfold measurements, waist and hip circumferences, and waist-hip (W–H) ratio can be found in Table 2. Overall, anthropometrics parameters, as well as nutrient and energy consumption based on the FFQ, did not change in intervention and placebo groups from baseline to follow-up (Online Resource 1). No intervention effects were observed, and effects did not depend on sex.

**Table 1:** Screening characteristics of overweight or obese older men and women with elevated SCF who completed the study (*n* = 40)

	All (*n* = 40)	Men (*n* = 23)	Women (*n* = 17)
Age (years)	69 ± 4	69 ± 4	68 ± 4
Height (cm)	172 ± 9	177 ± 7	165 ± 7
Weight (kg)	84.0 ± 9.7	86.7 ± 8.1	79.0 ± 10.2
BMI (kg/m^2^)	28.1 ± 2.6	27.7 ± 2.2	28.8 ± 3.1
CFQ, 0–100	48 ± 8	47 ± 7	50 ± 9
Systolic blood pressure (mmHg)	130 ± 12	131 ± 12	128 ± 13
Diastolic blood pressure (mmHg)	80 ± 8	81 ± 8	78 ± 10
Cholesterol (mmol/L)	5.9 ± 0.8	5.9 ± 0.9	6.1 ± 0.8
Triacylglycerol (mmol/L)	1.6 ± 1.0	1.5 ± 0.6	1.6 ± 1.4
Glucose (mmol/L)	5.6 ± 0.9	5.8 ± 0.5	5.3 ± 1.3

Values are means ± SDs

*BMI* body mass index, *CFQ* Cognitive Failure Questionnaire, *SCF* Subjective Cognitive Failures

**Table 2:** Anthropometrics at baseline and follow-up after a protein hydrolysate intervention versus a placebo group in a double-blind randomized controlled trial in adults with  elevated SCF

	Intervention (*n* = 20)			Placebo (*n* = 20)			*p* value^a^		
	Baseline	Follow-up	Mean difference	Baseline	Follow-up	Mean difference	Intervention effect [95% CI]	Sex × intervention	Intervention
Height (m)	172 ± 10	172 ± 10	0 ± 0	173 ± 8	173 ± 8	0 ± 0	0 [0, 0]	0.12	0.75
Weight (kg)	82.9 ± 10.4	83.3 ± 9.9	0.4 ± 1.8	85.1 ± 9.4	84.9 ± 10.5	−0.1 ± 2.2	0.5 [−0.8, 1.9]	0.58	0.36
BMI (kg/m^2^)	28.0 ± 2.8	28.2 ± 2.7	0.2 ± 0.6	28.3 ± 2.8	28.3 ± 3.4	0.0 ± 0.8	0.2 [−0.2, 0.6]	0.64	0.32
Body fat (%)	33 ± 7	34 ± 7	0 ± 2	34 ± 7	34 ± 7	0 ± 1	0 [−1, 1]	0.43	0.71
WC (cm)	99.8 ± 8.4	99.9 ± 8.4	0.0 ± 0.5	100.4 ± 8.3	100.4 ± 8.4	0.1 ± 0.6	0.0 [−0.4, 0.3]	0.22	0.79
HC (cm)	107 ± 6	107 ± 6	0 ± 0	108 ± 7	108 ± 7	0 ± 1	0 [0, 0]	0.60	0.99
W–H ratio	0.93 ± 0.07	0.93 ± 0.07	0.00 ± 0.01	0.93 ± 0.07	0.93 ± 0.07	0.00 ± 0.00	0.00 [0.00, 0.00]	0.92	0.63

Values are means ± SDs

*HC* hip circumference, *SCF* Subjective Cognitive Failures, *WC* waist circumference, *W–H ratio* waist-to-hip ratio

^a^An ANCOVA with baseline anthropometric values as a covariate and sex, intervention, and a sex × intervention interaction as fixed factors was first conducted. When the interaction term did not reach statistical significance, it was removed from the final model to estimate intervention effects. Intervention effects [95% CI] are reported after adjustment for baseline and sex

### Cognitive performance

**Fig. 2:**
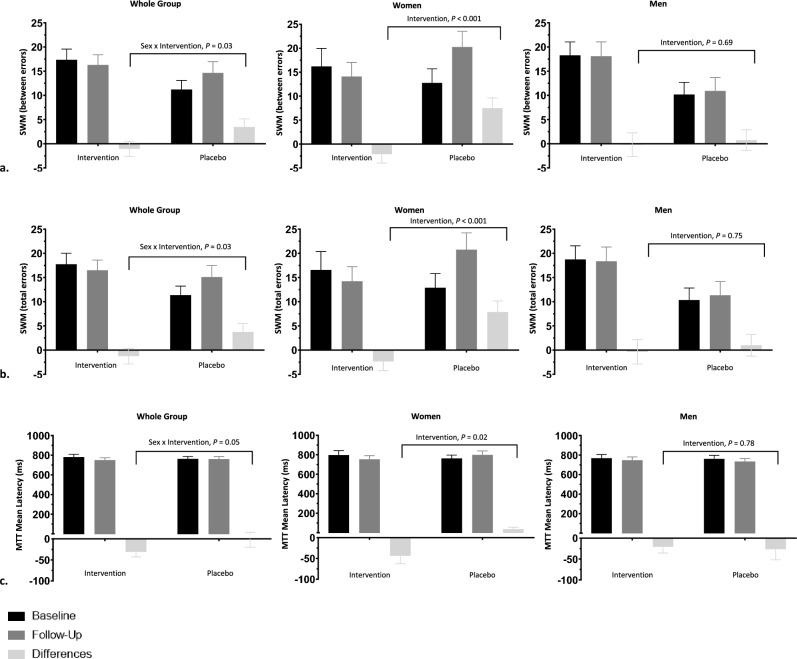
Changes in the cognitive performance SWM and MTT tasks for the whole group, and women and men separately after a protein hydrolysate intervention versus a placebo group in a double-blind randomized controlled trial in adults with elevated SCF. Values are means ± SEM. MTT, multitasking task; SCF, Subjective Cognitive Failures; SWM, spatial working memory. Tasks from baseline to follow-up in the placebo and intervention groups. **a** SWM, between errors **b** SWM, total errors **c** MTT reaction latency, ms

The outcomes of cognitive tests after the egg-protein hydrolysate (NWT-03) intervention compared to those after the placebo product are shown in Table 3. Overall, there were no significant intervention effects for any of the performed tasks encompassing the attention and psychomotor speed, executive function, and memory domains. However, a significant sex × intervention interaction effect was identified for the SWM executive function task. Additionally, the interaction for the MTT, the other executive function task, nearly reached statistical significance (*p* = 0.05). Specifically, more pronounced beneficial effects were observed in women as compared to men for the amount of between errors (*p* = 0.03) and total errors (*p* = 0.03) in the SWM task, and the reaction latency (*p* = 0.05) for the MTT task. Subsequently, analyses were repeated for men and women separately. A significant reduction of 9 (95% CI: −14 to −3; *p* < 0.001) for between errors and reduction of 9 (95% CI: −15 to −3; *p* < 0.001) for total errors on the SWM were observed in women, but not in men (Fig. 2). Likewise, women demonstrated a reduction of 74 ms in reaction latency on the MTT (95% CI: −134 to −15; *p* = 0.02), but this effect was not observed in men. Effects for the attention and psychomotor speed, and memory tasks did, however, not differ between men and women.

**Table 3:** Outcomes of psychomotor speed and executive function cognitive tasks after a protein hydrolysate intervention versus a placebo group in adouble-blind randomized controlled trial in adults with elevated SCF

	Intervention (*n* = 20)			Placebo (*n* = 20)			*p* value^a^		
	Baseline	Follow-up	Mean difference	Baseline	Follow-up	Mean difference	Intervention effect [95% CI]	Sex x intervention	Intervention
*Psychomotor speed*									
RTI movement time (ms)	309 ± 69	303 ± 81	−6 ± 48	307 ± 87	298 ± 71	−9 ± 51	−17 [−47, 14]	0.90	0.27
RTI reaction time (ms)	389 ± 36	399 ± 41	10 ± 23	386 ± 37	384 ± 38	−3 ± 28	−2 [−19, 14]	0.84	0.76
*Executive function*									
MTT incongruency cost (ms)	137 ± 80	131 ± 61	−5 ± 58	120 ± 57	107 ± 66	−13 ± 88	10 [−27, 48]	0.64	0.58
MTT multitasking cost (ms)	256 ± 128	201 ± 94	−55 ± 125	287 ± 180	255 ± 161	−32 ± 174	9 [−67, 86]	0.98	0.80
MTT mean latency (ms)	764 ± 121	741 ± 104	−24 ± 71	781 ± 115	775 ± 112	−6 ± 69		0.05	
MTT (total errors)	9 ± 12	9 ± 11	0 ± 11	12 ± 14	11 ± 15	−1 ± 13	−5 [−12, 2]	0.92	0.13
SWM (between errors)	14 ± 10	14 ± 10	0 ± 8	15 ± 10	17 ± 9	2 ± 7		0.03	
SWM (strategy score)	8 ± 3	8 ± 3	0 ± 2	8 ± 3	9 ± 2	1 ± 3	0 [−1, 2]	0.18	0.58
SWM (total errors)	14 ± 10	15 ± 10	0 ± 8	15 ± 10	17 ± 10	2 ± 8		0.03	
*Memory*									
DMS (% correct)	79 ± 13	85 ± 14	5 ± 19	80 ± 12	84 ± 9	4 ± 15	−2 [−11, 6]	0.91	0.57
PAL (1st attempt memory score)	9 ± 3	10 ± 4	1 ± 3	11 ± 3	12 ± 3	1 ± 3	0 [−2, 2]	0.49	0.73
PAL (total errors)	26 ± 14	23 ± 15	−3 ± 11	21 ± 13	18 ± 11	−4 ± 11	−5 [−6, 7]	0.88	0.88

Values are means ± SDs

*DMS* delayed matching to sample, *MTT* multitasking task, *PAL* paired associates learning, *RTI* reaction time task, *SCF* Subjective Cognitive Failures, *SWM* spatial working memory

^a^An ANCOVA with baseline cognitive performance values as a covariate and sex, intervention, and a sex × intervention interaction as fixed factors was first conducted. When the interaction term did not reach statistical significance, it was removed from the final model to estimate intervention effects. Intervention effects [95% CI] are reported after adjustment for baseline and sex

### Brain vascular function

**Fig. 3:**
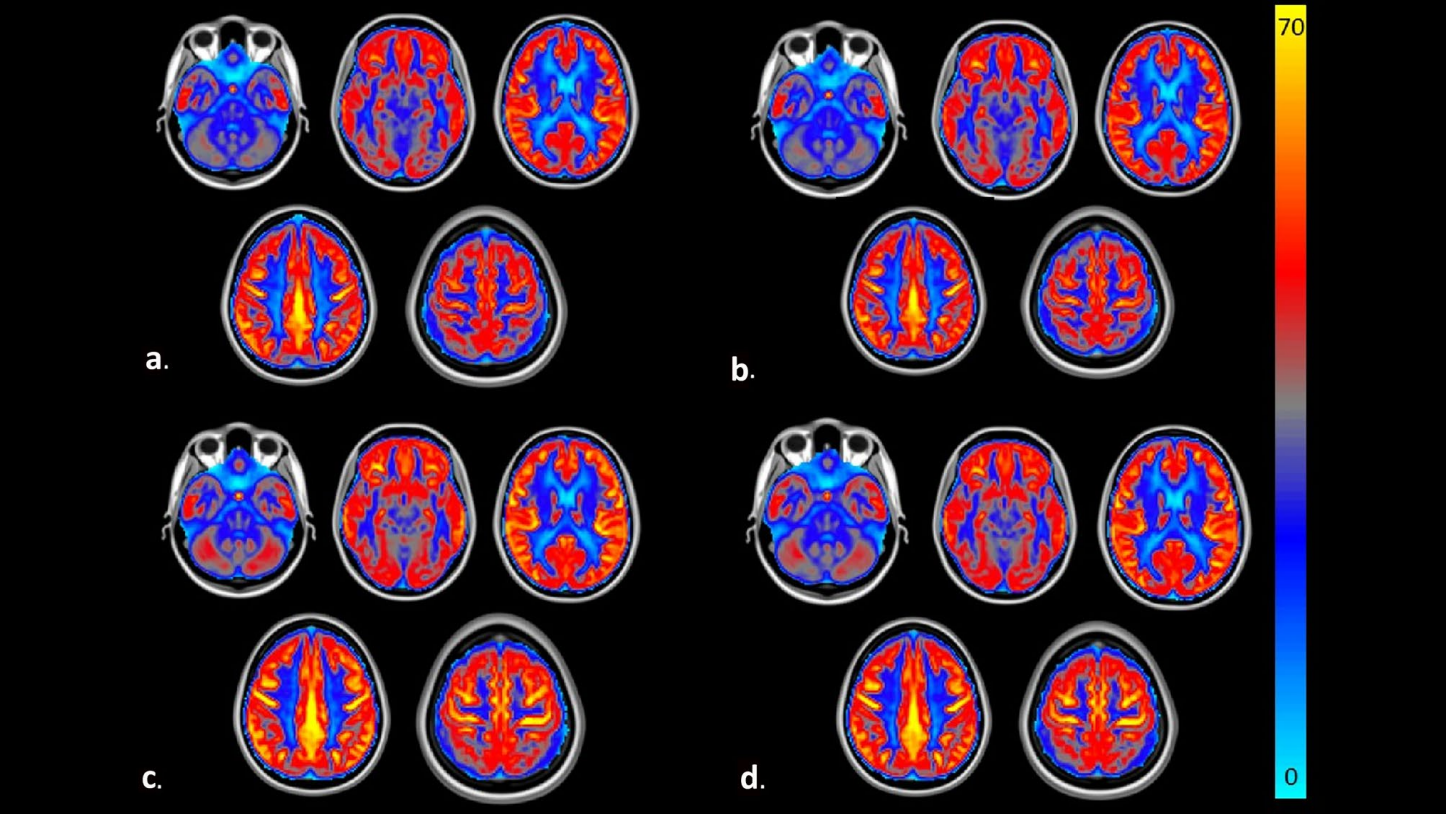
Pseudo-continuous arterial spin labeling (ASL) perfusion-weighted images which were acquired at the Scannexus research facility for a protein hydrolysate intervention versus a placebo group in a double-blind randomized controlled trial in adults with elevated SCF. The cerebral blood flow (CBF) is shown by the color bar in mL/70 g tissue/min. **a** Mean CBF map from the intervention (*n* = 19) at baseline **b** Mean CBF map from the intervention (*n* = 19) at follow-up **c** Mean CBF map from the placebo (*n* = 20) at baseline **d** Mean CBF map from the placebo (*n* = 20) at follow-up. SCF, subjective cognitive failures

As compared to the placebo, the NWT-03 intervention did not affect the whole-brain (*p* = 0.98), gray matter (*p* = 0.83), cortical (*p* = 0.99), and subcortical CBF (*p* = 0.92) (Table 4). Moreover, no significant sex × intervention interactions were observed, indicating that these results did not differ between men and women. Voxel-wise comparisons also did not reveal brain clusters that were differently affected between intervention and placebo groups. These comparisons were repeated for men and women separately, but study results did not differ. Visual representations of the average perfusion-weighted images at baseline and at follow-up for the intervention and placebo groups are depicted in Fig. 3.

**Table 4:** Outcomes of cerebral blood flow (CBF, ml/100 g brain tissue/min)^a^ and brain-derived neurotrophic factor (BDNF, pg/mL)^b^ after a protein hydrolysate intervention versus a placebo group in a double-blind randomized controlled trial in adults with elevated SCF

	Intervention			Placebo			*p* value^a^		
	Baseline	Follow-up	Mean difference	Baseline	Follow-up	Mean difference	Intervention effect [95% CI]	Sex × intervention	Intervention
*CBF (mL/100 g/min)*									
Whole-brain	35.2 ± 8.1	35.0 ± 8.7	−0.3 ± 4.3	38.6 ± 7.2	37.7 ± 6.3	−0.9 ± 5.2	−0.0 [−3.1, 3.0]	0.39	0.98
Gray matter	41.6 ± 10.2	40.8 ± 10.0	−0.7 ± 5.4	45.1 ± 8.3	43.9 ± 7.4	−1.2 ± 6.2	−0.7 [−4.4, 3.0]	0.39	0.83
Cortical	45.5 ± 11.0	45.0 ± 11.2	−0.5 ± 5.5	49.3 ± 8.6	48.0 ± 8.0	−1.3 ± 6.9	0.0 [−4.0, 4.0]	0.31	0.99
Subcortical	27.0 ± 8.4	26.4 ± 8.0	−0.6 ± 3.6	29.9 ± 9.1	28.9 ± 8.5	−1.0 ± 4.0	−0.1 [−2.5, 2.3]	0.38	0.92
*BDNF (pg/mL)*									
Serum concentrations	20,620 ± 6283	17,501 ± 6920	−3119 ± 10,046	21,896 ± 6471	19,754 ± 8717	−1,047 ± 13,132	−3272 [−9312, 2768]	0.62	0.31

*SCF* Subjective Cognitive Failures

^a^ [Values are means ± SDs; *n* = 39 (intervention = 19; placebo = 20)]

^b^ [Values are means ± SDs; *n* = 39 (intervention = 20; placebo = 19)]

^c^An ANCOVA with baseline CBF and BDNF values as a covariate and sex, intervention, and a sex × intervention interaction as fixed factors was first conducted. When the interaction term did not reach statistical significance, it was removed from the final model to estimate intervention effects. Intervention effects [95% CI] are reported after adjustment for baseline and sex

### Brain-derived neurotrophic factor

Changes in serum BDNF concentrations did not significantly differ between the intervention and placebo groups (*p* = 0.31). Additionally, effects did not depend on sex (*p* = 0.62; Table 4).

## Discussion

This double-blind, randomized, controlled parallel study has valuated the effects of the long-term consumption of the egg-protein hydrolysate NWT-03 on cognitive performance and brain vascular function in overweight or obese older adults with elevated SCF. Cognitive impairments are commonly associated with a higher risk of developing dementia [3] and frequently exhibits additional underlying metabolic risk factors, such as insulin resistance [40, 41] and impaired vascular function [42, 43]. Overall, no effects of NWT-03 were observed on cognitive performance tasks. However, for both executive function tasks (SWM and MTT), we observed more beneficial effects of the intervention in women, but not for men. Moreover, regional and voxel-wise CBF did not change, and these brain vascular function outcomes also did not differ between men and women. Additionally, no differences in serum BDNF concentrations, anthropometric measurements, and energy and nutrient intakes were found that could explain the observed changes on executive function, as these variables were comparable at baseline and did not change during the study.

Cognitive performance on tasks encompassing the attention and psychomotor speed, executive function, and memory domains did not change when all participants were analyzed from baseline to follow-up. However, after a significant sex × intervention interaction was observed, analyses were repeated for men and women separately. Improvements were observed in women for two independent executive functioning tasks. More specifically, in women, we observed a reduction in reaction latency on the MTT, along with fewer total errors and fewer between-task errors on the SWM. Improvements on the MTT task suggest that participants can multitask better by ignoring irrelevant information, while improvements in the SWM task suggest better working memory and ability to use a strategy to manipulate visuospatial information [44]. For the SWM task, the placebo group observed a worsening in cognitive performance, which could also be interpreted as NWT-03 providing a protective effect against deterioration. However, we would not expect that such a deterioration could already occur over 36-weeks, and this worsening would have also been expected to be observed in the MTT task assessing the same cognitive domain. These findings are in line with observed improvements on executive function in our prior short-term intervention study involving the consumption of a comparable amount of NWT-03 (5.0 g vs. 5.7 g in the current study) for 4 weeks [17]. In particular, a reduction in response time during the anti-cue paradigm was found, an executive function task which is analogous to the MTT and SWM tasks provided by CANTAB [18]. Therefore, our findings suggest that the short-term effects that we previously observed in adults with metabolic syndrome are maintained in the long-term for a higher-risk population of women with elevated SCF. People with subjective cognitive complaints are at a greater risk for developing Mild Cognitive Impairment (MCI) [45], where evidence already supports that 16% of MCI patients can reverse their diagnosis [46], also through non-pharmacological means [47]. This evidence highlights the potential clinical relevance of our study.

An important question remains as to why improvements on executive function were limited to women, rather than being observed in both sexes, as was noted in our previous study involving participants with metabolic syndrome [17]. Furthermore, we need to address the lack of impact on the attention and psychomotor speed and memory domains. Based on the slightly higher SCF experienced in women (mean = 50) compared to men (mean = 47), it may be hypothesized that the women may have had more room to benefit from a dietary intervention impacting executive function at this stage in their life. Therefore, a population of older men and women with elevated SCF cannot be directly compared to a population with younger individuals and metabolic syndrome. However, more research is needed to confirm or refute these speculations. In support, two soy studies, specifically targeting older women, have already demonstrated improvements on executive function [13, 14]. Similarly, Mohajeri and colleagues conducted an egg-protein hydrolysate intervention exclusively in middle-aged women, which also showed improvements on executive function [7]. These studies exemplify that women can benefit from dietary protein interventions, although it is unclear why in studies using a casein peptide [16], a peanut intervention [11], a multi-nutrient whey protein supplementation [12], and a high protein diet [15], results did not differ between women and men. However, apart from the egg-protein hydrolysate [7] and casein-derived peptide [16] studies, the other interventions contained non-protein components that do not allow us to completely attribute these beneficial effects to dietary proteins. Therefore, more RCTs on dietary proteins including both men and women which are adequately powered to detect sex differences are needed. Although underlying mechanisms are not entirely clear, it could be speculated that physiological differences between older men and women with elevated SCF play a role. In fact, it has been established that a combination of factors including hormonal changes due to menopause that also impact metabolism [48] cause faster cognitive decline in women compared to men [49]. This was particularly observed for executive function, but not for memory, and may be related to the fact that a decline on executive function is one of the first symptoms of dementia in women [50]. Furthermore, one observational study found significant associations between better cognitive function with plant and total protein intake in women, but not in men [51]. In a case–control study of patients with dementia and healthy controls, lower cognitive function in dementia patients was also associated with lower protein intake, but only in women [52].

NWT-03 has been shown to potentially improve peripheral vascular function [19] and cardiometabolic risk markers [20]. However, it still remained to be investigated whether these beneficial peripheral effects extend to the brain, as impaired brain vascular function is considered an important mechanism preceding the development of an impaired cognitive performance [21, 22]. Notably, CBF was not affected in this study, which indicates that other mechanisms may be involved for the cognitive performance benefits observed in women. Both a voxel-wise and regional approach to evaluate differences in CBF were used, the prior examining differences in the amount of CBF by comparing brain images pixel by pixel, whereas the latter uses the volume of the entire brain or specific brain regions based on an atlas [53]. Unlike previous dietary protein intervention studies that employed indirect techniques such as transcranial doppler ultrasound or near-infrared spectroscopy [11, 23, 25, 54], the utilization of an ASL voxel-wise approach allows for the detection of local differences that may have been previously overlooked [55]. Additionally, it can be pointed out that, although other studies in our lab have evaluated regional and voxel-wise CBF in older, overweight or obese populations using ASL [6, 56], the current study demonstrated comparatively lower baseline CBF values. This difference suggests that the participants’ brain vascular function, related to their elevated SCF status, may have declined below a threshold that makes it more challenging to influence CBF through protein hydrolysate interventions. Although CBF was not impacted, it is still possible that the small peptide components of NWT-03 crossed the blood–brain barrier (BBB) [57] and directly bound to receptors that modulate the release of neurotransmitters in specific brain regions [58], thereby improving executive function in women, without impacting brain vascular function. This possibility is supported by previous research on animal models that gene expression patterns and sex-specific genes can influence BBB permeability [59–61], resulting in potential differences in men and women’s peptide uptake. Alternatively, we can speculate that there may be a direct link between peripheral vascular function and cognitive performance, which may elucidate the observed effects on cognitive performance, without mediating effects of CBF. This could be through mechanisms impacting oxidative stress and inflammation, BBB integrity, neurotransmitter balance and brain insulin uptake [62–65]. Alternatively, changes in BDNF concentrations have been shown to improve cognition [66] through dietary interventions [67] by promoting synaptic plasticity and neurogenesis. However, no overall intervention effects were observed and, therefore, we can conclude that these improvements on executive function in women were not impacted by changes in serum BDNF. This is consistent with the findings observed in the 4-week study on NWT-03 [17], but we cannot eliminate the possibility that BDNF concentrations changed in the brain.

This double-blind, randomized, placebo-controlled parallel study had several strengths and weaknesses. First, the primary study outcomes were assessed using validated, reproducible methods to evaluate changes in brain vascular function [68, 69] and cognitive performance [44, 70–72]. In fact, the use of MRI with ASL [68] allows for a highly reproducible method of quantification of CBF [69]. To the best of our knowledge, this was also the first protein hydrolysate intervention to focus on a well-defined study population involving older adults with elevated SCF using a validated questionnaire [1, 26]. Finally, no study-related serious adverse events or protocol deviations were reported in the study diaries and the study product was well tolerated. One limitation of our study is its focus on a specific, homogenous target population, which may limit the generalizability of our findings. Additionally, while our study offers insights into cognitive performance outcomes and regional CBF differences, it underscores the importance of including sex as a consideration in the power calculations of future studies to further elucidate these effects. Finally, we do not have conclusive evidence of COVID-19 infections among our study participants throughout the duration of the study, and the randomization process in our study design was intended to mitigate any potential imbalance caused by undetected COVID-19 cases. However, we acknowledge that we cannot completely rule out the possibility that undetected or previous COVID-19 infections modified intervention effects observed following long-term consumption of NWT-03.

## Conclusion

Our data indicate that daily intake of 5.7 g NWT-03 for 36 weeks did not affect overall cognitive performance in an older population consisting of adults with elevated SCF, but evidence was provided for beneficial effects on parameters reflecting executive function in postmenopausal women as compared to men. There were no changes in the psychomotor speed or memory domains. In our previous 4-week NWT-03 study, changes on executive function were observed [17], which aligns with the current findings. Furthermore, this study expands on these results by demonstrating the long-term maintenance of the observed beneficial effects. However, no beneficial effects were found in regional and voxel-wise CBF using ASL, and the potentially differential results between men and women to an egg-protein hydrolysate intervention also still remains to be elucidated.

## Supplementary Information

Below is the link to the electronic supplementary material.

Supplementary file1 (XLSX 15 KB)

## Data Availability

The datasets produced and/or examined in the present study can be obtained upon a reasonable request from the corresponding author (P.J.J.).
